# Microscopy and Pathology

**Published:** 1881-10

**Authors:** J. J. M. Angear

**Affiliations:** Fort Madison, Iowa


### MICROSCOPY AND PATHOLOGY.

### FOR THOSE WHO HAVE NEITHER TIME NOR MONEY TO BECOME MICROSCOPISTS.

BY J. J. M. ANGEAR, A. M., M. D., FORT MADISON, IOWA.

Written for the Western Medical Reporter.

It is true that microscopy is a science, and, perhaps, that truth deters many a young medical man from putting himself in a position to see nature and understand what he reads.

He entertains the idea that a microscope costs a fortune, and in order to understand it, he must possess an extraordinary genius, and devote all his time to it to be able to achieve anything in the line of microscopy—all of which is false, and we wish to remove as far as possible these erroneous and damaging impressions. Ordinary intelligence, with a determined purpose, and a few dollars, is what is needed.

Want of time is the excuse of many, and these very men are too frequently found on a dry-goods box at the corner of the street, or some frequented place, either listening to, or telling, stories of questionable purity for hours together, and yet they have no time to spend with the microscope.

"Have'n't the money to spare" is another cry we frequently hear. Watch and see if you do not misappropriate money enough annually for follies—for that which is not bread—for that which profiteth not. To be plain, we mean for drinks which beclouds, blights, enfeebles and ruins, also the accursed weed that contaminates, disgusts and enslaves. Open your eyes. Take a pencil and make your own mathematical calculation, and see if you have not willingly spent more time and money for that which is worse than useless than the majority of first-class microscopists have in making themselves such.

We do not desire to enter the *morale* of the question, nor the finance, any further than to tear off the flimsy gauze, so that all may see for themselves whether it be reality or a mere phantom. A word to the wise is sufficient. Cease to do evil; learn to do well. Save your money; economize your time; become a microscopist; strike boldly for the front of your profession.

To become a diagnostician you must become a microscopist. We will give two simple, but practical cases from Bennett's Clinical Lectures, p. 76:

A child was supposed to be affected with worms, because it passed in abundance yellowish shreds, which, to the naked eye, closely resembled ascarides. All kinds of vermifuge remedies had been tried in vain. On examining the shreds with a microscope, I found them to consist of undigested spiral vessels of plants; and they ceased to appear when the vegetable broth used as food was abandoned.

An individual was supposed to be laboring under dysentery, from the frequent passage of the yellow pulpy masses in the stools, accompanied with tormina and other symptoms. On examining these masses with the microscope, I found them to consist of undigested potato skins. On inquiry it was ascertained that this person had eaten the skins with the potatoes. On causing these to be removed before dinner the alarming appearance ceased, and the other symptoms also disappeared.

In our next issue we will try and help those who need help, and are willing to help themselves, by giving some very elementary instruction.—*The Western Medical Reporter.*

